# Comparative Clinical Effectiveness and Operating Efficiency of Different Ultrasonic Devices for Performance of Sleeve Gastrectomy

**DOI:** 10.1007/s11695-026-08892-y

**Published:** 2026-08-18

**Authors:** Monali Misra, I-Wen Pan, Zasim Azhar Siddiqui, Frank Bisceglie, Matthew Kroh

**Affiliations:** 1 Cedars-Sinai Medical Center, Los Angeles, CA USA; 2 Surgical OU, Clinical Medical Affair and HEPR, Medtronic, Minneapolis, MN USA; 3 Cleveland Clinic Lerner College of Medicine, Cleveland, OH USA; 4 Surgical OU, Core Surgical Innovations, Medtronic, Minneapolis, MN USA

**Keywords:** Sleeve gastrectomy, Advanced energy devices, Operating room time

## Abstract

**Introduction:**

This study aims to evaluate clinical and economic outcomes of using different ultrasonic energy devices in patients undergoing sleeve gastrectomy (SG).

**Methods:**

The patient cohort was extracted from PINC AI™ Healthcare Data 2019–2023, including individuals aged ≥ 18 who underwent primary elective gastric sleeve procedures using either corded (CD) or cordless (CL) ultrasonic devices (UD). Two analysis groups were evaluated: total SG regardless of the - stapler brand used in the operation (Cohort I), and UD used with an identifiable same-brand stapler in the operation (Cohort II). A propensity score matching (PSM) method with a caliper of 0.2 and 1:1 nearest-neighbor matching was performed for patients who used CD and CL. We applied multivariable generalized linear model (GLM) analyses for sensitivity analyses.

**Results:**

24160 and 18906 patients were included for cohorts I (82.3% CD and 17.7% CL; 4268 PSM paired) and II (81.7% CD and 18.3% CL; 3282 PSM paired), respectively. CD had higher operating-room time (minutes) than CL in both Cohort I (mean difference: 27 (95% confidence interval (CI): (24, 29), *p* < 0.001) and II (27 (24, 30), *p* < 0.001). There is no difference in incidence rates of blood transfusion, bleeding, and 30-day readmission, and total inpatient cost and length of stay between CD and CL. The sensitivity analyses showed similar results.

**Conclusions:**

The study shows that CL had similar clinical and economic outcomes to CD, and CL was more efficient in patients treated with sleeve gastrectomy.

**Key Points:**

• Cordless ultrasonic devices were more efficient when used in sleeve gastrectomy.

• Outcomes of sleeve gastrectomy used cordless/corded ultrasonic device were similar.

• Resource use of cordless/corded ultrasonic device was similar in sleeve gastrectomy.

**Supplementary Information:**

The online version contains supplementary material available at https://doi.org/10.1007/s11695-026-08892-y.

## Introduction

Advanced energy devices for vessel sealing were developed to improve the safety and efficiency of surgical procedures, which could reduce blood loss and shorten operative time [1]. The evolution of ultrasonic technology into multifunctional surgical devices in the 1990s represented a major advancement in minimally invasive surgery, allowing integration of cutting, dissection, coagulation, and tissue grasping within a single system. Corded Harmonic Scalpel™ was the first developed ultrasonic energy device in 1990 [1, 2]. Later, Sonicision™ cordless ultrasonic dissection device was launched in 2012. These devices provide rapid hemostasis with a favorable safety profile by generating high-frequency mechanical vibration that denatures proteins by disrupting hydrogen bonds, forming a coagulum, and enabling simultaneous tissue division and vessel sealing [3, 4]. They also offer safety advantages by reducing lateral thermal tissue injury and tissue charring through lower peak thermal generation [5, 6]. Additionally, these devices improve visualization during minimally invasive procedures by reducing surgical plume formation [7]. 

While ultrasonic devices have demonstrated favorable patient outcomes compared to the conventional approach, clinical studies comparing corded (CD) and cordless (CL) ultrasonic devices (UDs) have shown similar clinical performance, with no significant differences in operative time, blood loss, dissection failure, hospital stay, or complication rates in sleeve gastrectomy and radical gastrectomy procedures [8, 9]. However, cordless ultrasonic devices have been shown to offer ergonomic advantages, including improved maneuverability and faster assembly and setup during surgery [8]. 

Laparoscopic sleeve gastrectomy is now the predominant bariatric procedure in the U.S., yet no U.S.-based studies have compared CD and CL ultrasonic devices in this setting. A study conducted outside the U.S. comparing CD and CL in SG is limited by small sample sizes, limiting the generalizability of its findings [8, 9]. Thus, we conducted a real-world evidence analysis using a multi-health system U.S. database to provide more generalizable evidence on the comparative effectiveness of CD and CL ultrasonic energy devices. Accordingly, the objective of this study is to evaluate the clinical and economic outcomes associated with the use of different ultrasonic energy devices in SG.

## Methods

### Data Sources

The PINC AI™ Healthcare Data (PHD) comprises U.S. hospital-based, service-level information on inpatient discharge [10]. More than 1,400 hospitals/healthcare systems contribute data to the PHD, with more than 9 million visits annually since 2012, representing approximately 25% of annual United States inpatient admissions. The PHD contains information on hospital and visit characteristics, admitting and attending physician specialties, healthcare payers, and patient data, including demographics, disease states, diagnoses, costs, medications, and device details from hospital discharge billing files. The charge master and hospital charge files, which allow us to identify surgical devices through text search. The PHD is de-identified by the HIPAA Privacy Rule. This study was determined exempt from full board review by Sterling IRB.

### Cohort Selection and Setting

Data was extracted from PHD 2019–2023. Patients who underwent elective primary minimally invasive (robotic-assisted or laparoscopic) sleeve gastrectomy in an inpatient setting, age ≥ 18, no missing age or gender information, with inpatient cost > 0, who used either of the cordless (CL, Sonicision™) or corded (CD, Harmonic™) ultrasonic energy devices (UDs) were included. We excluded patients who used multiple energy devices or devices not studied.

### Intervention

**Fig. 1:**
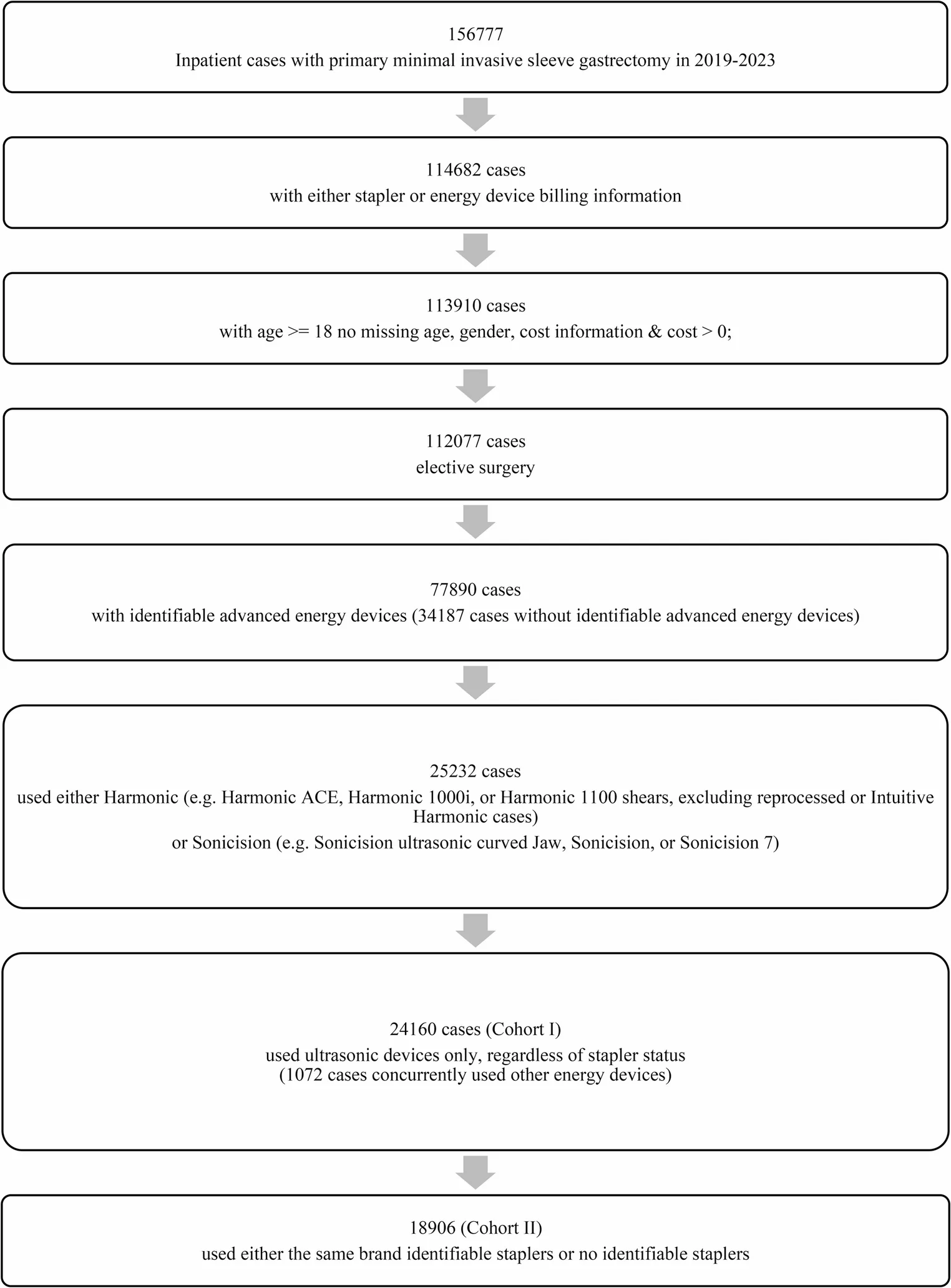
Cohort selection diagram

Patients who used either corded (CD, J&J Harmonic™) or cordless (CL, Medtronic Sonicision™) ultrasonic devices (UDs) in sleeve gastrectomy. Products were identified in the hospital charge master file via text search, using keywords (“Harmonic”, “Sonicision”, or product name) and catalog numbers to improve accuracy. The detailed product inclusion and exclusion are shown in Fig. 1.

### Outcomes Measurement

Clinical outcomes included complications **(**blood transfusion, bleeding), 30-day readmission, and intensive care unit (ICU) visits. We used ICD-10 diagnosis and procedure codes and Current Procedural Terminology (CPT) codes to identify the incidence of complications in patients’ inpatient claim files [11–13]. Bleeding events include patients who had a secondary diagnosis coded as acute posthemorrhagic anemia, hemorrhage, melena, or postprocedural hemorrhage of a digestive system organ or structure following a digestive system procedure. The billing codes indicated that patients who underwent blood transfusion procedures or received blood products, such as platelets, red blood cells, or whole blood, were classified as having a blood transfusion. Details of the codes used are provided in the supplementary. Patients were classified as having an ICU visit if at least one ICU-related visit and its corresponding charge were recorded.

Healthcare resource utilization, including operating room time (ORT) in minutes, costs, and lengths of stay (LOS), was evaluated. Total hospitalization costs and LOS (in days) were obtained from the Premier “PATDEMO” file; all costs were converted to 2023 U.S. dollars using the consumer price index for hospital services [14]. ORT in minutes was obtained from the charge master file, which includes the time from the patient’s entry into the operating room to their exit.

### Patient Characteristics

Patient characteristics were extracted from “PATDEMO” file, including age (dichotomized to < 65, ≥65), gender (male, female), race (non-Hispanic White, others), payor (private commercial payer, others), diagnosis (obesity: yes, no), Charlson comorbidity index scores [15] (CCI, dichotomized to CCI 0–2, CCI 3+), robotic platform use (yes, no), hiatal hernia repair (yes, no) [16, 17], and use of stapling (yes/no). Charlson comorbidity index scores, robotic platform use, and concurrent hiatal hernia repair were identified using ICD-10 diagnosis or procedure codes, or CPT codes. (see supplementary). In addition, patients’ hospital charges containing the keyword “robotic” were classified as involving the robotic platform.

### Provider Characteristics

The providers’ characteristics included: census region (Northeast, Midwest, South, and West); hospital location (urban or rural); bed size (less than 500 beds or 500 or more beds); teaching status (teaching or non-teaching hospital); and provider volume. Provider’s volume (hospital and surgeon) was grouped into three categories based on annual bariatric procedure volume: both high volume, defined as treatment at facilities where both the hospital and the surgeon had case volumes above the 75th percentile of all providers; either high volume, defined as either the hospital or the surgeon had volumes above the 75th percentile; and both low volume, defined as treatment at facilities where both the hospital and the surgeon had volumes at or below the 75th percentile.

### Statistical Analysis

All analyses were conducted in two cohorts: Cohort I included cases utilizing corded or cordless ultrasonic energy devices regardless of stapler identification or stapler brand, whereas Cohort II was restricted to cases utilizing corded or cordless ultrasonic energy devices with the same identifiable stapler brand or without an identifiable stapler. Propensity scores (PS) were calculated using logistic regression including patients’ and providers’ characteristics. We conducted 1-to-1 nearest-neighbor matching without replacement between CL (treated) and CD (controlled) ultrasonic device groups using a caliper width of 0.2 on the propensity score scale. Absolute standardized mean differences for all covariates were calculated before and after matching to assess covariate balance. The post-PS matching outcome estimate was obtained using bias-corrected regression adjustment (double-robust adjustment) [6]. All analyses were conducted using SAS 9.4 and Stata 18.5, employing two-sided statistical tests. P-values less than 0.05 were considered statistically significant.

## Sensitivity Analysis

To test the robustness of the results, we used multivariable generalized linear models (GLMs) with binomial or Gamma distributions for binary and continuous outcomes, respectively, and a log-link function. The covariates adjusted for in the GLMs included patients’ and providers’ characteristics. Post-GLM marginal effects for outcome estimation were reported (adjusted outcomes).

## Results

### Baseline Patient and Provider Characteristics

Based on the study criteria, a total of 24,160 and 18,906 sleeve gastrectomy cases were identified in Cohort I (any stapler brand) and Cohort II (same stapler brand as UD), respectively, representing cases using ultrasonic devices regardless of stapler brand and those using ultrasonic devices with same-brand staplers (Fig. 1). In Cohort I, 4,268 (17.7%) cases used CL, whereas in Cohort II, 3,464 (18.3%) cases used CL UD. In both cohorts, the use of identifiable staplers was more common in CD than in CL (Cohort I: 78.8% vs. 72.0%; Cohort II: 72.6% vs. 65.5%). Most SG cases in both cohorts were performed among patients younger than 65 years, female, and diagnosed with obesity. CL was more commonly used in urban settings in both cohorts (CL vs. CD: Cohort I: 96.1% vs. 87.3%; Cohort II: 95.3% vs. 84.2%) compared with CD, and in hospitals with < 500 beds (CL vs. CD: Cohort I: 80.4% vs. 66.6%; Cohort II: 76.9% vs. 71.5%). Patients who used CL were more likely to be treated by high-volume providers than those who used CD (CL vs. CD: Cohort I, 66.8% vs. 59.3%; Cohort II, 73.7% vs. 59.7%). Table 1 presents the differences in baseline patient and provider characteristics between the two ultrasonic device groups.

**Table 1:** Baseline patients and providers’ characteristics

	Cohort I: All cases with either CD or CL				Cohort II: All cases with either CD or CL, with the same-brand identifiable staplers			
	Total *N* (%)	CL *N* (%)	CD *N* (%)	*P*-value	Total *N* (%)	CL *N* (%)	CD *N* (%)	*P*-value
Total	24,160 (100)	4,268 (17.7)	19,892 (82.3)		18,906 (100)	3,464 (18.3)	15,442 (81.7)	
Stapler use								
No	5,419 (22.4)	1,194 (28.0)	4,225 (21.2)	< 0.001	5,419 (28.7)	1,194 (34.5)	4,225 (27.4)	< 0.001
Yes	18,741 (77.6)	3,074 (72.0)	15,667 (78.8)		13,487 (71.3)	2,270 (65.5)	11,217 (72.6)	
Robotic platform								
No	23,663 (97.9)	4,237 (99.3)	19,426 (97.7)	< 0.001	18,629 (98.5)	3,460 (99.9)	15,169 (98.2)	< 0.001
Yes	497 (2.1)	31 (0.7)	466 (2.3)		277 (1.5)	4 (0.1)	273 (1.8)	
Hiatal hernia repair								
No	19,314 (79.9)	3,473 (81.4)	15,841 (79.6)	0.010	14,916 (78.9)	2,918 (84.2)	11,998 (77.7)	< 0.001
Yes	4,846 (20.1)	795 (18.6)	4,051 (20.4)		3,990 (21.1)	546 (15.8)	3,444 (22.3)	
Age group								
< 65	22,993 (95.2)	4,103 (96.1)	18,890 (95.0)	0.001	18,003 (95.2)	3,337 (96.3)	14,666 (95.0)	0.001
65+	1,167 (4.8)	165 (3.9)	1,002 (5.0)		903 (4.8)	127 (3.7)	776 (5.0)	
Gender								
Female	19,835 (82.1)	3,425 (80.3)	16,410 (82.5)	0.001	15,551 (82.3)	2,785 (80.4)	12,766 (82.7)	0.002
Male	4,325 (17.9)	843 (19.8)	3,482 (17.5)		3,355 (17.8)	679 (19.6)	2,676 (17.3)	
Married status								
Others	12,895 (53.4)	2,320 (54.4)	10,575 (53.2)	0.155	10,088 (53.4)	1,947 (56.2)	8,141 (52.7)	< 0.001
Married	11,265 (46.6)	1,948 (45.6)	9,317 (46.8)		8,818 (46.6)	1,517 (43.8)	7,301 (47.3)	
Race								
Others	12,956 (53.6)	2,970 (69.6)	9,986 (50.2)	0.001	10,078 (53.3)	2,419 (69.8)	7,659 (49.6)	< 0.001
White	11,204 (46.4)	1,298 (30.4)	9,906 (49.8)		8,828 (46.7)	1,045 (30.2)	7,783 (50.4)	
Payer								
Others	10,952 (45.3)	1,948 (45.6)	9,004 (45.3)	0.671	8,508 (45.0)	1,633 (47.1)	6,875 (44.5)	0.005
Private	13,208 (54.7)	2,320 (54.4)	10,888 (54.7)		10,398 (55.0)	1,831 (52.9)	8,567 (55.5)	
Obesity diagnosis								
No	908 (3.8)	148 (3.5)	760 (3.8)	0.269	773 (4.1)	93 (2.7)	680 (4.4)	< 0.001
Yes	23,252 (96.2)	4,120 (96.5)	19,132 (96.2)		18,133 (95.9)	3,371 (97.3)	14,762 (95.6)	
Comorbidities								
0–2	22,668 (93.8)	3,966 (92.9)	18,702 (94.0)	0.007	17,754 (93.9)	3,235 (93.4)	14,519 (94.0)	0.159
3+	1,492 (6.2)	302 (7.1)	1,190 (6.0)		1,152 (6.1)	229 (6.6)	923 (6.0)	
Provider region								
Northeast	4,404 (18.2)	1,017 (23.8)	3,387 (17.0)	< 0.001	2,289 (12.1)	977 (28.2)	1,312 (8.5)	< 0.001
Midwest	6,564 (27.2)	1,729 (40.5)	4,835 (24.3)		5,573 (29.5)	1,461 (42.2)	4,112 (26.6)	
South	9,451 (39.1)	288 (6.8)	9,163 (46.1)		7,795 (41.2)	251 (7.3)	7,544 (48.9)	
West	3,741 (15.5)	1,234 (28.9)	2,507 (12.6)		3,249 (17.2)	775 (22.4)	2,474 (16.0)	
Provider location								
Rural	2,685 (11.1)	165 (3.9)	2,520 (12.7)	< 0.001	2,596 (13.7)	163 (4.7)	2,433 (15.8)	< 0.001
Urban	21,475 (88.9)	4,103 (96.1)	17,372 (87.3)		16,310 (86.3)	3,301 (95.3)	13,009 (84.2)	
Provider bed size								
<500 beds	16,683 (69.1)	3,431 (80.4)	13,252 (66.6)	< 0.001	13,705 (72.5)	2,663 (76.9)	11,042 (71.5)	< 0.001
500+	7,477 (31.0)	837 (19.6)	6,640 (33.4)		5,201 (27.5)	801 (23.1)	4,400 (28.5)	
Provider teaching status								
No	10,871 (45.0)	1,918 (44.9)	8,953 (45.0)	0.915	8,718 (46.1)	1,366 (39.4)	7,352 (47.6)	< 0.001
Yes	13,289 (55.0)	2,350 (55.1)	10,939 (55.0)		10,188 (53.9)	2,098 (60.6)	8,090 (52.4)	
Provider (hospital and surgeon) bariatric procedure volume								
Both high volume	14,653 (60.7)	2,851 (66.8)	11,802 (59.3)	< 0.001	11,771 (62.3)	2,553 (73.7)	9,218 (59.7)	< 0.001
Either high volume	5,028 (20.8)	614 (14.4)	4,414 (22.2)		3,839 (20.3)	418 (12.1)	3,421 (22.2)	
Both low volume	4,479 (18.5)	803 (18.8)	3,676 (18.5)		3,296 (17.4)	493 (14.2)	2,803 (18.2)	
Surgical year								
2019	6,639 (27.5)	1,129 (26.5)	5,510 (27.7)	< 0.001	5,409 (28.6)	878 (25.4)	4,531 (29.3)	< 0.001
2020	4,504 (18.6)	750 (17.6)	3,754 (18.9)		3,728 (19.7)	612 (17.7)	3,116 (20.2)	
2021	4,966 (20.6)	904 (21.2)	4,062 (20.4)		3,804 (20.1)	774 (22.3)	3,030 (19.6)	
2022	4,627 (19.2)	768 (18.0)	3,859 (19.4)		3,563 (18.9)	642 (18.5)	2,921 (18.9)	
2023	3,424 (14.2)	717 (16.8)	2,707 (13.6)		2,402 (12.7)	558 (16.1)	1,844 (11.9)	

### Clinical Outcomes and Healthcare Resource Utilization

Before matching, the unadjusted outcomes showed a lower incidence of ICU visits (Cohort I: 0.3% vs. 0.6%, *p* = 0.012; Cohort II: 0.3% vs. 0.7%, *p* = 0.014) and a shorter mean ORT (mean (standard deviation)) in minutes: Cohort I:109 (40) vs. 123 (46), *p* < 0.001; Cohort II: 109 (41) vs. 120 (46), *p* < 0.001) for procedures involving CL compared with CD in both cohorts. Conversely, total costs were higher for CL, while no other outcomes demonstrated statistically significant differences. Table 2 summarizes the unadjusted comparisons between the two UD groups.

**Table 2:** Unadjusted clinical outcomes and healthcare resource utilization by corded and cordless ultrasonic devices

	Cohort I			Cohort II		
Clinical outcomes						
	CD *N* (%)	CL *N* (%)	*p*-value	CD *N* (%)	CL *N* (%)	*p*-value
Blood transfusion	90 (0.5%)	16 (0.4%)	0.49	76 (0.5%)	14 (0.4%)	0.50
Bleeding	185 (0.9%)	42 (1.0%)	0.74	154 (1.0%)	36 (1.0%)	0.82
30 days readmission	351 (1.8%)	75 (1.8%)	0.97	267 (1.7%)	61 (1.8%)	0.90
ICU visit	124 (0.6%)	13 (0.3%)	**0.012**	111 (0.7%)	12 (0.3%)	**0.014**
Healthcare resource utilization						
	CD mean (STD)	CL mean (STD)	p-value	CD mean (STD)	CL mean (STD)	p-value
Total costs (2023 USD)	$13,029 ($5,544)	$13,703 ($7,288)	**< 0.001**	$13,051 ($5,465)	$13,542 ($7,681)	**< 0.001**
Total costs (2023 USD), median (IQR)	$12,104 ($9,838-$14,786)	$13,117 ($10,949-$15,431)	< 0.001	$12,027 ($9,872-$14,673)	$13,024 ($10,784-$15,318)	< 0.001
Operating room time (minutes)	123 (46)	109 (40)	**< 0.001**	120 (46)	109 (41)	**< 0.001**
Length of stay (days)	1 (0.9)	1 (1.7)	0.50	1 (0.9)	1 (1.9)	0.40

Abbreviations: * CD *corded ultrasonic device, *CL *cordless ultrasonic device, *N* number of cases, *STD *standard deviation, *IQR *interquartile range

**Fig. 2:**
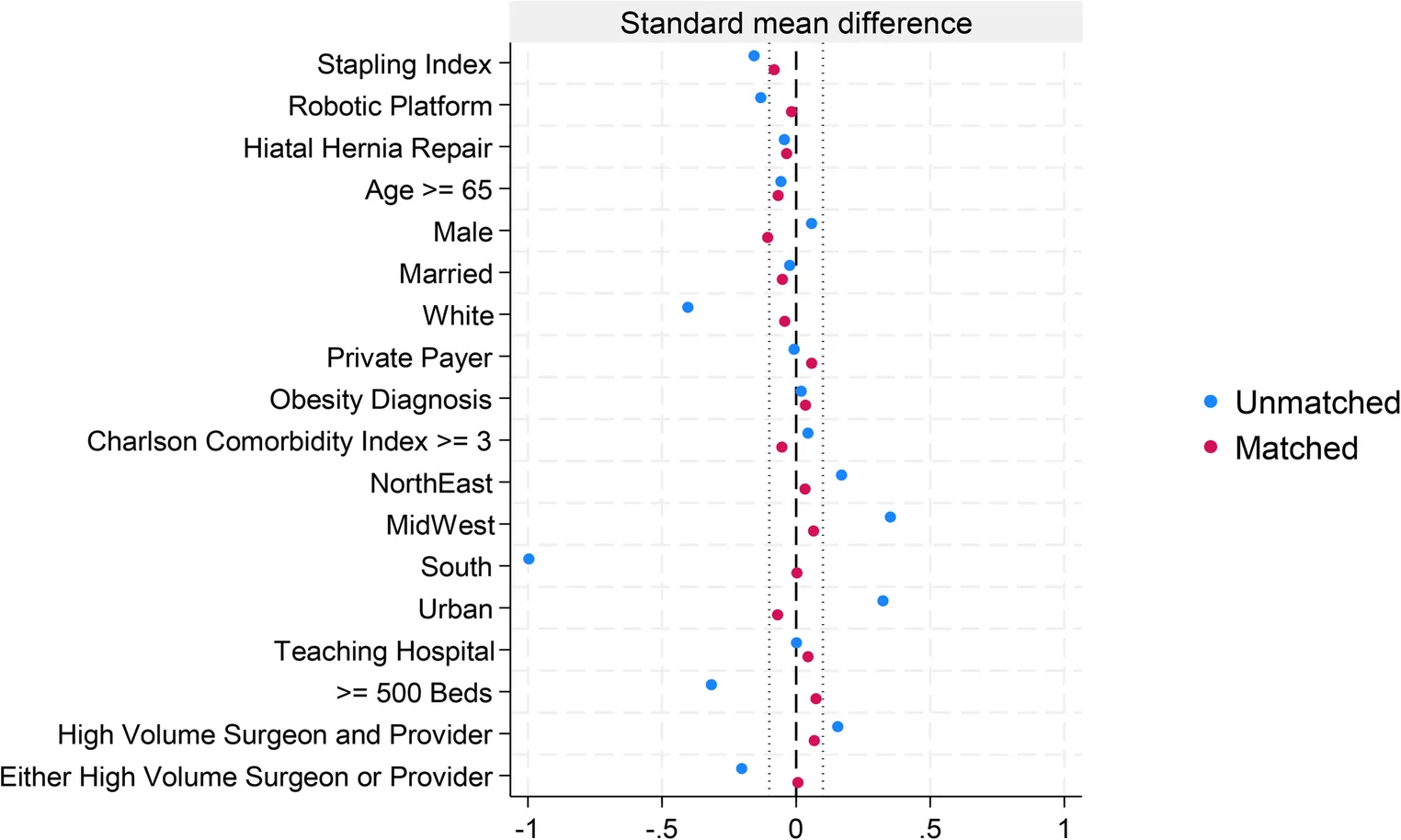
The covariate balance between CD and CL for cohort I, unmatched and propensity-score matched

**Fig. 3:**
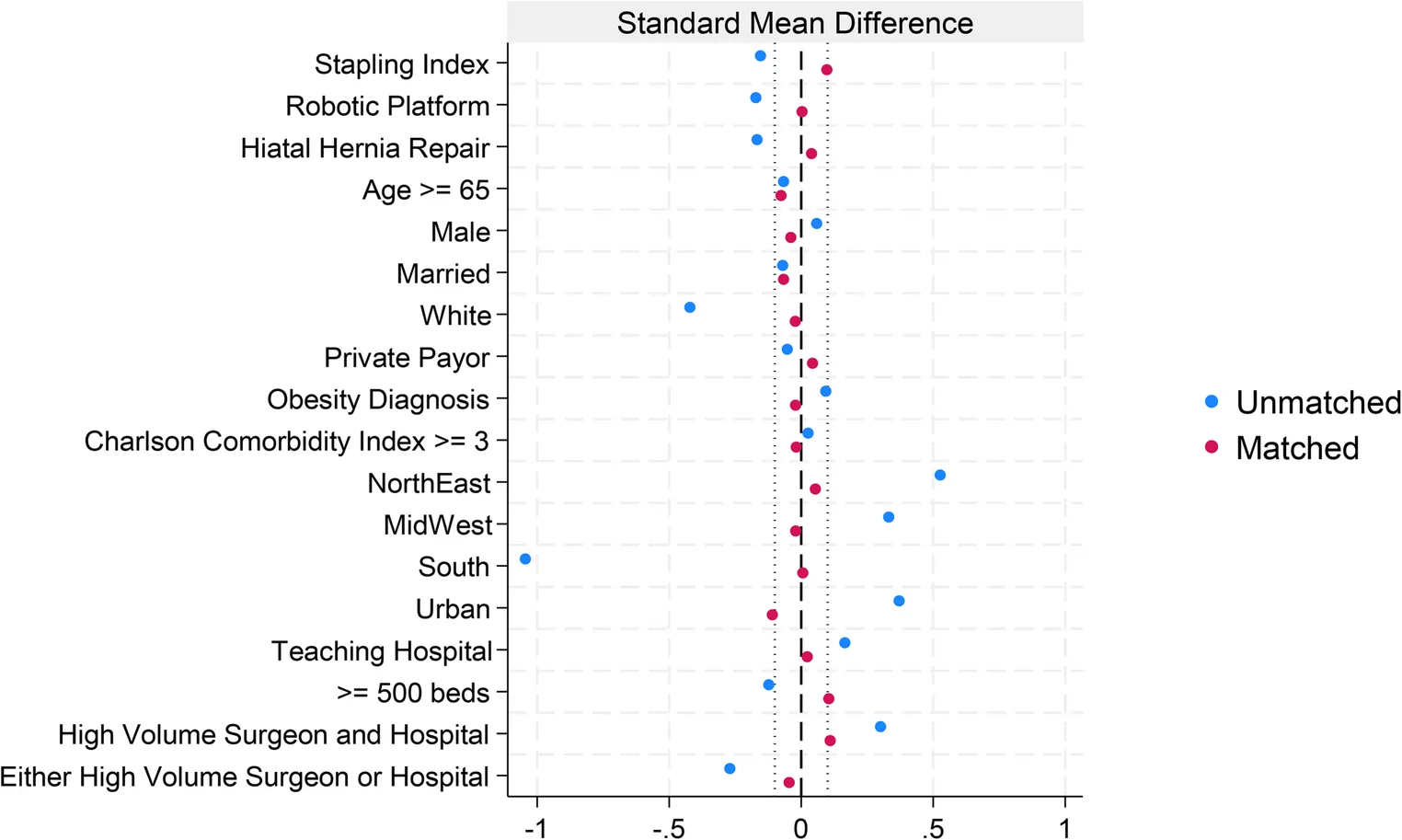
The covariate balance between CD and CL for cohort II, unmatched and propensity-score matched

Propensity score matching resulted in 4,268 and 3,282 matched pairs of SG cases for Cohort I and Cohort II, respectively **(**Fig. 2 and Fig. 3**)**. The matched analyses yielded findings consistent with the unadjusted results, patients treated with CL showed a lower rate of ICU visits (incidence rate: Cohort I: 0.3% vs. 0.7%, *p* = 0.015; Cohort II: 0.3% vs. 1.1%, *p* = 0.001) and reduced mean ORT (mean (standard error) in minutes: Cohort I: 109 (0.62) vs. 136 (0.89), *p* < 0.001; Cohort II: 110 (0.75) vs. 136 (1.19), *p* < 0.001) compared with CD. Meanwhile, all other outcomes, including total costs, showed no significant difference between groups. Table 3 shows the matched-adjusted comparisons between the two ultrasonic device groups. Adjusted differences for the evaluated outcomes are shown in the supplementary.

**Table 3:** Adjusted propensity score matched clinical outcomes and healthcare resource utilization by corded and cordless ultrasonic devices

	Cohort I			Cohort II		
Clinical outcomes						
	CD %	CL %	*p*-value	CD %	CL %	*p*-value
Blood transfusion	0.4	0.4	0.97	0.5	0.4	0.83
Bleeding	1.1	1.0	0.48	1.2	1.1	0.64
30 days readmission	1.7	1.8	0.94	1.5	1.7	0.59
ICU visit	0.7	0.3	**0.015**	1.1	0.3	**0.001**
Healthcare resource utilization						
	CD mean (SE)	CL mean (SE)	p-value	CD mean (SE)	CL mean (SE)	p-value
Total costs (2023 USD)	$13,719 ($61)	$13,703 ($112)	0.903	$13,401 ($78)	$13,554 ($137)	0.332
Operating room time (minutes)	136 (0.89)	109 (0.62)	**< 0.001**	136 (1.19)	110 (0.75)	**< 0.001**
Length of stay (days)	1 (0.01)	1 (0.03)	0.989	1 (0.01)	1 (0.03)	0.236

Abbreviations: *CD* corded ultrasonic devices, *CL* cordless ultrasonic devices, *SE *standard error

### Sensitivity Analysis

Sensitivity analysis using a multivariable GLM showed fewer ICU visits among CL cases than among CD cases (Cohort I: 0.3% vs. 0.6%, *p* = 0.005; Cohort II: 0.4% vs. 0.7%, *p* = 0.038). Consistent with the PSM findings, mean ORT was lower for CL than CD (mean (standard error) in minutes: Cohort I: 108 (0.66) vs. 123 (0.32), *p* < 0.001; Cohort II: 111 (0.84) vs. 120 (0.36), *p* < 0.001). (see Supplementary)

## Discussions

The current study explored the performance of two popular ultrasonic energy devices and showed no difference in clinical outcomes between patients who were treated with the two devices for sleeve gastrectomy, except that those treated with corded devices had longer ORT and higher incidence of intensive care unit visits. The study demonstrated that cordless ultrasonic devices were likely to be more efficient in sleeve gastrectomy, while providing comparable safety features and total hospitalization costs.

Sleeve gastrectomy is the most commonly performed bariatric procedure worldwide, with laparoscopic SG accounting for the majority of cases in the United States [18, 19]. This procedure requires precise tissue dissection and effective hemostasis to minimize bleeding and perioperative complications. Ultrasonic energy devices facilitate these requirements by supporting vessel sealing and tissue dissection while maintaining hemostasis and visualization during minimally invasive surgery. Ultrasonic devices are also associated with improved clinical outcomes compared with conventional surgical techniques across multiple surgical procedures, including gastrectomy in patients with gastric cancer, where these devices were associated with reduced operative time and intraoperative blood loss compared with conventional hemostatic methods [11, 20]. 

The choice of energy devices from the radio-frequency or ultrasonic hybrid devices used in SG relies on the surgeon’s decision-making, based on their experiences and preferences. When ultrasonic devices are used, cordless devices may offer greater benefits than corded/wired devices. The performance of ultrasonic energy devices has been evaluated by bleeding events and LOS [11–13]. Prior studies have reported no clinically significant differences between corded and cordless ultrasonic devices in sleeve gastrectomy and radical gastrectomy procedures, though the findings restrict the generalizability due to small sample size [8, 9]. In this study, we compared incidence rates of bleeding and blood transfusion between patients who used CL and CD. The adjusted rates were almost identical, further supporting the findings and showing no clinically significant differences between patients treated with CL and CD in SG. However, we found significant differences in ORT between the two ultrasonic devices during sleeve gastrectomy, regardless of whether the devices were used with or without an identifiable same-brand stapler. One study [21] had shown that the usage time of energy devices during laparoscopic gastrectomy for cancer was about 57% of the total time (skin-to-skin), while the usage time of the stapler was only 3.5% to 4.7%. Although we were unable to account for all types of activities and device usage time, the study may provide insight to support our findings. The reduced operative time was likely associated with the use of energy devices, i.e., patients treated with CL. This is unsurprising, as previous studies have found that cordless ultrasonic devices are installed more quickly, easier to assemble, more maneuverable, and easier to use than wired devices [1, 8, 9, 22]. Another potential reason for the ORT reduction may be attributed to the surgical plume. An experimental study previously conducted to compare plume obstruction using ultrasonic devices in bovine liver, including Harmonic ACE™ (CD) and Sonicision™ (CL), showed that CL produced significantly fewer plumes than CD [23]. The ultrasonic devices used rely on heat and pressure to cut tissue and coagulate blood vessels during the procedure, generating a plume that obscures vision, interrupts surgery, and pauses the procedure, which may increase ORT if the plume does not evacuate. However, given the physiological and anatomical differences, a direct translation of outcomes from an animal model experiment to human clinical outcomes may not be feasible. Also, the study was conducted in 2012. With recent technological advances, the role of the surgical plume of both devices in reducing ORT is unclear and warrants further investigation.

In this study, we observed significant differences in ICU visits between the CL and CD groups. However, the clinical importance and impact of these findings are unknown and would presumably not be attributable to case-efficiency improvements with cordless devices. 

### Limitations

This retrospective study was conducted using a hospital claim database, but not a randomized clinical study. It has limitations due to the nature of the claim and billing data. First, the procedure types and complications were defined using ICD-10 diagnosis and procedure codes or CPT codes. The hospital is reportedly submitting the codes for reimbursement; this is likely reliable. However, validating coding remains a challenge. Secondly, the text search product from the hospital charges may be misclassified if the hospital did not enter the information correctly, for example, did not enter the correct name of the product, or misspelled the product number. Third, total inpatient costs did not directly reflect device acquisition costs and were influenced by factors such as hospital purchasing contracts, bundled pricing arrangements, supply chain agreements, and institutional cost-accounting practices. Also, although the energy device may not be the primary medical device used in SG and the usage time of the energy devices in SG may be shorter than prior observations in gastric cancer surgery, it is still used a significant percentage of total time and a critical part of the operation; the observed outcomes may be associated or contributed by both the ultrasonic device and the staplers used, including stapler type, staple-line reinforcement, and institutional device utilization practices. In addition, ORT may be affected by institutional workflow, room setup, and room turnover, which were not observed in the study and may introduce bias and variability in the estimation. This is a limitation of the dataset. Lastly, the authors’ conflict of interest, which may introduce a potential bias, has been disclosed in this manuscript. Further constructed clinical trials may provide better insight into these findings.

## Conclusions

In this real-world analysis of sleeve gastrectomy, cordless ultrasonic devices were shown to be associated with shorter operating room time while demonstrating comparable rates of perioperative complications, readmissions, length of stay, and total inpatient costs compared with corded devices. This technology may offer efficiency advantages without compromising clinical or economic outcomes, though prospective studies may accurately define outcomes.

## Supplementary Information

Below is the link to the electronic supplementary material.

Supplementary Material 1

## Data Availability

The data that support the findings of this study are available from Premier at https://premierinc.com/, but restrictions apply to the availability of these data, which were used under license for the current study, and so are not publicly available. Data are however available from the authors upon reasonable request and with permission of Premier.
