# Correction: Comparative Clinical Effectiveness and Operating Efficiency of Different Ultrasonic Devices for Performance of Sleeve Gastrectomy

**DOI:** 10.1007/s11695-026-08911-y

**Published:** 2026-08-21

**Authors:** Monali Misra, I-Wen Pan, Zasim Azar Siddqui, Frank Bisceglie, Matthew Kroh

**Affiliations:** 1 Cedars-Sinai Medical Center, Los Angeles, CA USA; 2 Surgical OU, Clinical Medical Affair and HEPR, Medtronic, Minneapolis, MN USA; 3 Cleveland Clinic Lerner College of Medicine, Cleveland, OH USA; 4 Surgical OU, Core Surgical Innovations, Medtronic, Minneapolis, MN USA

**Correction: Obesity Surgery**

**https://doi.org/10.1007/s11695-026-08892-y**

The first author's name is Monali Misra.

