# Supplementary material for: Comparative Clinical Effectiveness and Operating Efficiency of Different Ultrasonic Devices for Performance of Sleeve Gastrectomy
Source: Obes Surg. 2026 Aug 18;36(10):5173–82. doi: 10.1007/s11695-026-08892-y (PMC13645881; doi:10.1007/s11695-026-08892-y)
Supplement: Supplementary file 1 [file 11695_2026_8892_MOESM1_ESM.docx]

Supplementary

**Table S1: Study codes**

Blood transfusion

**ICD-10-PCS**

| 30233H0 | Transfusion of Autologous Whole Blood into Peripheral Vein, Percutaneous Approach |
| --- | --- |
| 30233N0 | Transfusion of autologous Red blood cells into peripheral vein, percutaneous approach |
| 30243H0 | Transfusion of Autologous Whole Blood into Central Vein, Percutaneous Approach |
| 30243N0 | Transfusion of Autologous Red Blood Cells into Central vein, percutaneous approach |
| 30233H1 | Transfusion of Nonautologous Whole Blood into Peripheral Vein, Percutaneous Approach |
| 30243H1 | Transfusion of Nonautologous Whole Blood into Central vein, percutaneous approach |
| 30233N1 | Transfusion of Nonautologous Red Blood Cells into Peripheral Vein, Percutaneous Approach |
| 30233P1 | Transfusion of Nonautologous Frozen Red Cells into Peripheral Vein, Percutaneous Approach |
| 30243N1 | Transfusion of Nonautologous Red Blood Cells into Central vein, percutaneous approach |
| 30243P1 | Transfusion of Nonautologous Frozen Red Cells into Central vein, percutaneous approach |
| 30230H0 | Transfusion of Autologous Whole Blood into Peripheral Vein, Open Approach |
| 30230H1 | Transfusion of Nonautologous Whole Blood into Peripheral Vein, Open Approach |
| 30230J0 | Transfusion of Autologous Serum Albumin into Peripheral Vein, Open Approach |
| 30230J1 | Transfusion of Nonautologous Serum Albumin into Peripheral Vein, Open Approach |
| 30230K0 | Transfusion of Autologous Frozen Plasma into Peripheral Vein, Open Approach |
| 30230K1 | Transfusion of Nonautologous Frozen Plasma into Peripheral Vein, Open Approach |
| 30230L0 | Transfusion of Autologous Fresh Plasma into Peripheral Vein, Open Approach |
| 30230L1 | Transfusion of Nonautologous Fresh Plasma into Peripheral Vein, Open Approach |
| 30230M0 | Transfusion of Autologous Plasma Cryoprecipitate into Peripheral Vein, Open Approach |
| 30230M1 | Transfusion of Nonautologous Plasma Cryoprecipitate into Peripheral Vein, Open Approach |
| 30230N0 | Transfusion of Autologous Red Blood Cells into Peripheral Vein, Open Approach |
| 30230N1 | Transfusion of Nonautologous Red Blood Cells into Peripheral Vein, Open Approach |
| 30230P0 | Transfusion of Autologous Frozen Red Cells into Peripheral Vein, Open Approach |
| 30230P1 | Transfusion of Nonautologous Frozen Red Cells into Peripheral Vein, Open Approach |
| 30230Q0 | Transfusion of Autologous White Cells into Peripheral Vein, Open Approach |
| 30230Q1 | Transfusion of Nonautologous White Cells into Peripheral Vein, Open Approach |
| 30230R0 | Transfusion of Autologous Platelets into Peripheral Vein, Open Approach |
| 30230R1 | Transfusion of Nonautologous Platelets into Peripheral Vein, Open Approach |
| 30230S0 | Transfusion of Autologous Globulin into Peripheral Vein, Open Approach |
| 30230T0 | Transfusion of Autologous Fibrinogen into Peripheral Vein, Open Approach |
| 30230V0 | Transfusion of Autologous Antihemophilic Factors into Peripheral Vein, Open Approach |
| 30230W0 | Transfusion of Autologous Factor IX into Peripheral Vein, Open Approach |
| 30233J0 | Transfusion of Autologous Serum Albumin into Peripheral Vein, Percutaneous Approach |
| 30233J1 | Transfusion of Nonautologous Serum Albumin into Peripheral Vein, Percutaneous Approach |
| 30233K0 | Transfusion of Autologous Frozen Plasma into Peripheral Vein, Percutaneous Approach |
| 30233K1 | Transfusion of Nonautologous Frozen Plasma into Peripheral Vein, Percutaneous Approach |
| 30233L0 | Transfusion of Autologous Fresh Plasma into Peripheral Vein, Percutaneous Approach |
| 30233L1 | Transfusion of Nonautologous Fresh Plasma into Peripheral Vein, Percutaneous Approach |
| 30233M0 | Transfusion of Autologous Plasma Cryoprecipitate into Peripheral Vein, Percutaneous Approach |
| 30233M1 | Transfusion of Nonautologous Plasma Cryoprecipitate into Peripheral Vein, Percutaneous Approach |
| 30233P0 | Transfusion of Autologous Frozen Red Cells into Peripheral Vein, Percutaneous Approach |
| 30233Q0 | Transfusion of Autologous White Cells into Peripheral Vein, Percutaneous Approach |
| 30233Q1 | Transfusion of Nonautologous White Cells into Peripheral Vein, Percutaneous Approach |
| 30233R0 | Transfusion of Autologous Platelets into Peripheral Vein, Percutaneous Approach |
| 30233R1 | Transfusion of Nonautologous Platelets into Peripheral Vein, Percutaneous Approach |
| 30233S0 | Transfusion of Autologous Globulin into Peripheral Vein, Percutaneous Approach |
| 30233T0 | Transfusion of Autologous Fibrinogen into Peripheral Vein, Percutaneous Approach |
| 30233V0 | Transfusion of Autologous Antihemophilic Factors into Peripheral Vein, Percutaneous Approach |
| 30233W0 | Transfusion of Autologous Factor IX into Peripheral Vein, Percutaneous Approach |
| 30240H0 | Transfusion of Autologous Whole Blood into Central Vein, Open Approach |
| 30240H1 | Transfusion of Nonautologous Whole Blood into Central Vein, Open Approach |
| 30240J0 | Transfusion of Autologous Serum Albumin into Central Vein, Open Approach |
| 30240J1 | Transfusion of Nonautologous Serum Albumin into Central Vein, Open Approach |
| 30240K0 | Transfusion of Autologous Frozen Plasma into Central Vein, Open Approach |
| 30240K1 | Transfusion of Nonautologous Frozen Plasma into Central Vein, Open Approach |
| 30240L0 | Transfusion of Autologous Fresh Plasma into Central Vein, Open Approach |
| 30240L1 | Transfusion of Nonautologous Fresh Plasma into Central Vein, Open Approach |
| 30240M0 | Transfusion of Autologous Plasma Cryoprecipitate into Central Vein, Open Approach |
| 30240M1 | Transfusion of Nonautologous Plasma Cryoprecipitate into Central Vein, Open Approach |
| 30240N0 | Transfusion of Autologous Red Blood Cells into Central Vein, Open Approach |
| 30240N1 | Transfusion of Nonautologous Red Blood Cells into Central Vein, Open Approach |
| 30240P0 | Transfusion of Autologous Frozen Red Cells into Central Vein, Open Approach |
| 30240P1 | Transfusion of Nonautologous Frozen Red Cells into Central Vein, Open Approach |
| 30240Q0 | Transfusion of Autologous White Cells into Central Vein, Open Approach |
| 30240Q1 | Transfusion of Nonautologous White Cells into Central Vein, Open Approach |
| 30240R0 | Transfusion of Autologous Platelets into Central Vein, Open Approach |
| 30240R1 | Transfusion of Nonautologous Platelets into Central Vein, Open Approach |
| 30240S0 | Transfusion of Autologous Globulin into Central Vein, Open Approach |
| 30240T0 | Transfusion of Autologous Fibrinogen into Central Vein, Open Approach |
| 30240V0 | Transfusion of Autologous Antihemophilic Factors into Central Vein, Open Approach |
| 30240W0 | Transfusion of Autologous Factor IX into Central Vein, Open Approach |
| 30243J0 | Transfusion of Autologous Serum Albumin into Central Vein, Percutaneous Approach |
| 30243J1 | Transfusion of Nonautologous Serum Albumin into Central Vein, Percutaneous Approach |
| 30243K0 | Transfusion of Autologous Frozen Plasma into Central Vein, Percutaneous Approach |
| 30243K1 | Transfusion of Nonautologous Frozen Plasma into Central Vein, Percutaneous Approach |
| 30243L0 | Transfusion of Autologous Fresh Plasma into Central Vein, Percutaneous Approach |
| 30243L1 | Transfusion of Nonautologous Fresh Plasma into Central Vein, Percutaneous Approach |
| 30243M0 | Transfusion of Autologous Plasma Cryoprecipitate into Central Vein, Percutaneous Approach |
| 30243M1 | Transfusion of Nonautologous Plasma Cryoprecipitate into Central Vein, Percutaneous Approach |
| 30243P0 | Transfusion of Autologous Frozen Red Cells into Central Vein, Percutaneous Approach |
| 30243Q0 | Transfusion of Autologous White Cells into Central Vein, Percutaneous Approach |
| 30243Q1 | Transfusion of Nonautologous White Cells into Central Vein, Percutaneous Approach |
| 30243R0 | Transfusion of Autologous Platelets into Central Vein, Percutaneous Approach |
| 30243R1 | Transfusion of Nonautologous Platelets into Central Vein, Percutaneous Approach |
| 30243S0 | Transfusion of Autologous Globulin into Central Vein, Percutaneous Approach |
| 30243T0 | Transfusion of Autologous Fibrinogen into Central Vein, Percutaneous Approach |
| 30243V0 | Transfusion of Autologous Antihemophilic Factors into Central Vein, Percutaneous Approach |
| 30243W0 | Transfusion of Autologous Factor IX into Central Vein, Percutaneous Approach |
| 30250H0 | Transfusion of Autologous Whole Blood into Peripheral Artery, Open Approach |
| 30250H1 | Transfusion of Nonautologous Whole Blood into Peripheral Artery, Open Approach |
| 30250J0 | Transfusion of Autologous Serum Albumin into Peripheral Artery, Open Approach |
| 30250J1 | Transfusion of Nonautologous Serum Albumin into Peripheral Artery, Open Approach |
| 30250K0 | Transfusion of Autologous Frozen Plasma into Peripheral Artery, Open Approach |
| 30250K1 | Transfusion of Nonautologous Frozen Plasma into Peripheral Artery, Open Approach |
| 30250L0 | Transfusion of Autologous Fresh Plasma into Peripheral Artery, Open Approach |
| 30250L1 | Transfusion of Nonautologous Fresh Plasma into Peripheral Artery, Open Approach |
| 30250M0 | Transfusion of Autologous Plasma Cryoprecipitate into Peripheral Artery, Open Approach |
| 30250M1 | Transfusion of Nonautologous Plasma Cryoprecipitate into Peripheral Artery, Open Approach |
| 30250N0 | Transfusion of Autologous Red Blood Cells into Peripheral Artery, Open Approach |
| 30250N1 | Transfusion of Nonautologous Red Blood Cells into Peripheral Artery, Open Approach |
| 30250P0 | Transfusion of Autologous Frozen Red Cells into Peripheral Artery, Open Approach |
| 30250P1 | Transfusion of Nonautologous Frozen Red Cells into Peripheral Artery, Open Approach |
| 30250Q0 | Transfusion of Autologous White Cells into Peripheral Artery, Open Approach |
| 30250Q1 | Transfusion of Nonautologous White Cells into Peripheral Artery, Open Approach |
| 30250R0 | Transfusion of Autologous Platelets into Peripheral Artery, Open Approach |
| 30250R1 | Transfusion of Nonautologous Platelets into Peripheral Artery, Open Approach |
| 30250S0 | Transfusion of Autologous Globulin into Peripheral Artery, Open Approach |
| 30250T0 | Transfusion of Autologous Fibrinogen into Peripheral Artery, Open Approach |
| 30250V0 | Transfusion of Autologous Antihemophilic Factors into Peripheral Artery, Open Approach |
| 30250W0 | Transfusion of Autologous Factor IX into Peripheral Artery, Open Approach |
| 30253H0 | Transfusion of Autologous Whole Blood into Peripheral Artery, Percutaneous Approach |
| 30253H1 | Transfusion of Nonautologous Whole Blood into Peripheral Artery, Percutaneous Approach |
| 30253J0 | Transfusion of Autologous Serum Albumin into Peripheral Artery, Percutaneous Approach |
| 30253J1 | Transfusion of Nonautologous Serum Albumin into Peripheral Artery, Percutaneous Approach |
| 30253K0 | Transfusion of Autologous Frozen Plasma into Peripheral Artery, Percutaneous Approach |
| 30253K1 | Transfusion of Nonautologous Frozen Plasma into Peripheral Artery, Percutaneous Approach |
| 30253L0 | Transfusion of Autologous Fresh Plasma into Peripheral Artery, Percutaneous Approach |
| 30253L1 | Transfusion of Nonautologous Fresh Plasma into Peripheral Artery, Percutaneous Approach |
| 30253M0 | Transfusion of Autologous Plasma Cryoprecipitate into Peripheral Artery, Percutaneous Approach |
| 30253M1 | Transfusion of Nonautologous Plasma Cryoprecipitate into Peripheral Artery, Percutaneous Approach |
| 30253N0 | Transfusion of Autologous Red Blood Cells into Peripheral Artery, Percutaneous Approach |
| 30253N1 | Transfusion of Nonautologous Red Blood Cells into Peripheral Artery, Percutaneous Approach |
| 30253P0 | Transfusion of Autologous Frozen Red Cells into Peripheral Artery, Percutaneous Approach |
| 30253P1 | Transfusion of Nonautologous Frozen Red Cells into Peripheral Artery, Percutaneous Approach |
| 30253Q0 | Transfusion of Autologous White Cells into Peripheral Artery, Percutaneous Approach |
| 30253Q1 | Transfusion of Nonautologous White Cells into Peripheral Artery, Percutaneous Approach |
| 30253R0 | Transfusion of Autologous Platelets into Peripheral Artery, Percutaneous Approach |
| 30253R1 | Transfusion of Nonautologous Platelets into Peripheral Artery, Percutaneous Approach |
| 30253S0 | Transfusion of Autologous Globulin into Peripheral Artery, Percutaneous Approach |
| 30253T0 | Transfusion of Autologous Fibrinogen into Peripheral Artery, Percutaneous Approach |
| 30253V0 | Transfusion of Autologous Antihemophilic Factors into Peripheral Artery, Percutaneous Approach |
| 30253W0 | Transfusion of Autologous Factor IX into Peripheral Artery, Percutaneous Approach |
| 30260H0 | Transfusion of Autologous Whole Blood into Central Artery, Open Approach |
| 30260H1 | Transfusion of Nonautologous Whole Blood into Central Artery, Open Approach |
| 30260J0 | Transfusion of Autologous Serum Albumin into Central Artery, Open Approach |
| 30260J1 | Transfusion of Nonautologous Serum Albumin into Central Artery, Open Approach |
| 30260K0 | Transfusion of Autologous Frozen Plasma into Central Artery, Open Approach |
| 30260K1 | Transfusion of Nonautologous Frozen Plasma into Central Artery, Open Approach |
| 30260L0 | Transfusion of Autologous Fresh Plasma into Central Artery, Open Approach |
| 30260L1 | Transfusion of Nonautologous Fresh Plasma into Central Artery, Open Approach |
| 30260M0 | Transfusion of Autologous Plasma Cryoprecipitate into Central Artery, Open Approach |
| 30260M1 | Transfusion of Nonautologous Plasma Cryoprecipitate into Central Artery, Open Approach |
| 30260N0 | Transfusion of Autologous Red Blood Cells into Central Artery, Open Approach |
| 30260N1 | Transfusion of Nonautologous Red Blood Cells into Central Artery, Open Approach |
| 30260P0 | Transfusion of Autologous Frozen Red Cells into Central Artery, Open Approach |
| 30260P1 | Transfusion of Nonautologous Frozen Red Cells into Central Artery, Open Approach |
| 30260Q0 | Transfusion of Autologous White Cells into Central Artery, Open Approach |
| 30260Q1 | Transfusion of Nonautologous White Cells into Central Artery, Open Approach |
| 30260R0 | Transfusion of Autologous Platelets into Central Artery, Open Approach |
| 30260R1 | Transfusion of Nonautologous Platelets into Central Artery, Open Approach |
| 30260S0 | Transfusion of Autologous Globulin into Central Artery, Open Approach |
| 30260T0 | Transfusion of Autologous Fibrinogen into Central Artery, Open Approach |
| 30260V0 | Transfusion of Autologous Antihemophilic Factors into Central Artery, Open Approach |
| 30260W0 | Transfusion of Autologous Factor IX into Central Artery, Open Approach |
| 30263H0 | Transfusion of Autologous Whole Blood into Central Artery, Percutaneous Approach |
| 30263H1 | Transfusion of Nonautologous Whole Blood into Central Artery, Percutaneous Approach |
| 30263J0 | Transfusion of Autologous Serum Albumin into Central Artery, Percutaneous Approach |
| 30263J1 | Transfusion of Nonautologous Serum Albumin into Central Artery, Percutaneous Approach |
| 30263K0 | Transfusion of Autologous Frozen Plasma into Central Artery, Percutaneous Approach |
| 30263K1 | Transfusion of Nonautologous Frozen Plasma into Central Artery, Percutaneous Approach |
| 30263L0 | Transfusion of Autologous Fresh Plasma into Central Artery, Percutaneous Approach |
| 30263L1 | Transfusion of Nonautologous Fresh Plasma into Central Artery, Percutaneous Approach |
| 30263M0 | Transfusion of Autologous Plasma Cryoprecipitate into Central Artery, Percutaneous Approach |
| 30263M1 | Transfusion of Nonautologous Plasma Cryoprecipitate into Central Artery, Percutaneous Approach |
| 30263N0 | Transfusion of Autologous Red Blood Cells into Central Artery, Percutaneous Approach |
| 30263N1 | Transfusion of Nonautologous Red Blood Cells into Central Artery, Percutaneous Approach |
| 30263P0 | Transfusion of Autologous Frozen Red Cells into Central Artery, Percutaneous Approach |
| 30263P1 | Transfusion of Nonautologous Frozen Red Cells into Central Artery, Percutaneous Approach |
| 30263Q0 | Transfusion of Autologous White Cells into Central Artery, Percutaneous Approach |
| 30263Q1 | Transfusion of Nonautologous White Cells into Central Artery, Percutaneous Approach |
| 30263R0 | Transfusion of Autologous Platelets into Central Artery, Percutaneous Approach |
| 30263R1 | Transfusion of Nonautologous Platelets into Central Artery, Percutaneous Approach |
| 30263S0 | Transfusion of Autologous Globulin into Central Artery, Percutaneous Approach |
| 30263T0 | Transfusion of Autologous Fibrinogen into Central Artery, Percutaneous Approach |
| 30263V0 | Transfusion of Autologous Antihemophilic Factors into Central Artery, Percutaneous Approach |
| 30263W0 | Transfusion of Autologous Factor IX into Central Artery, Percutaneous Approach |

**CPT**

| 36430 | Transfusion blood and blodd products |
| --- | --- |
| 86890 | Under Transfusion Medicine Procedures |
| 86891 | Under Transfusion Medicine Procedures |
| 86985 | Under Transfusion Medicine Procedures |
| P9010 | Blood (whole), for transfusion, per unit |
| P9011 | Blood, split unit |
| P9012 | Cryoprecipitate, each unit |
| P9016 | Red blood cells, leukocytes reduced, each unit |
| P9017 | Fresh frozen plasma (single donor), frozen within 8 hours of collection, each unit |
| P9019 | Platelets, each unit |
| P9020 | Platelet rich plasma, each unit |
| P9021 | Red blood cells, each unit |
| P9022 | Red blood cells, washed, each unit |
| P9023 | Plasma, pooled multiple donor, solvent/detergent treated, frozen, each unit |
| P9032 | Platelets, irradiated, each unit |
| P9033 | Platelets, leukocytes reduced, irradiated, each unit |
| P9034 | Platelets, pheresis, each unit |
| P9035 | Platelets, pheresis, leukocytes reduced, each unit |
| P9036 | Platelets, pheresis, irradiated, each unit |
| P9037 | Platelets, pheresis, leukocytes reduced, irradiated, each unit |
| P9038 | Red blood cells, irradiated, each unit |
| P9039 | Red blood cells, deglycerolized, each unit |
| P9040 | Red blood cells, leukocytes reduced, irradiated, each unit |
| P9043 | Infusion, plasma protein fraction |
| P9044 | Plasma, cryoprecipitate reduced, each unit |
| P9048 | Infusion, plasma protein fraction (human), 5%, 250 ml |
| P9050 | Granulocytes, pheresis, each unit |
| P9051 | Whole blood or red blood cells, leukocytes reduced, cmv-negative, each unit |
| P9052 | Platelets, hla-matched leukocytes reduced, apheresis/pheresis, each unit |
| P9053 | Platelets, pheresis, leukocytes reduced, cmv-negative, irradiated, each unit |
| P9054 | Whole blood or red blood cells, leukocytes reduced, frozen, deglycerol, washed, each unit |
| P9055 | Platelets, leukocytes reduced, cmv-negative, apheresis/pheresis, each unit |
| P9056 | Whole blood, leukocytes reduced, irradiated, each unit |
| P9057 | Red blood cells, frozen/deglycerolized/washed, leukocytes reduced, irradiated, each unit |
| P9058 | Red blood cells, leukocytes reduced, cmv-negative, irradiated, each unit |
| P9059 | Fresh frozen plasma between 8-24 hours of collection, each unit |
| P9060 | Fresh frozen plasma, donor retested, each unit |
| P9070 | Plasma, pooled multiple donor, pathogen reduced, frozen, each unit |
| P9071 | Plasma (single donor), pathogen reduced, frozen, each unit |
| P9072 | Platelets, pheresis, pathogen reduced or rapid bacterial tested, each unit |

Bleeding

**ICD-10-CM**

| D62 | Acute posthemorrhagic anemia |
| --- | --- |
| R58 | Hemorrhage, not elsewhere classified |
| K92.1 | Melena (Black stools that occur as a result of gastrointestinal bleeding) |
| K92.2 | Gastrointestinal hemorrhage, unspecified |
| K91.840 | Postprocedural hemorrhage of a digestive system organ or structure following a digestive system procedure |
| K91.841 | Postprocedural hemorrhage of a digestive system organ or structure following other procedure |
| K91.61 | Intraoperative hemorrhage and hematoma of a digestive system organ or structure complicating a digestive system procedure |
| K91.62 | Intraoperative hemorrhage and hematoma of a digestive system organ or structure complicating other procedure |
| K91.870 | Postprocedural hematoma of a digestive system organ or structure following a digestive system procedure |
| K91.871 | Postprocedural hematoma of a digestive system organ or structure following other procedure |

**Table S2:**

**Main analysis: post-propensity score matching -adjusted differences of outcomes between CD and CL**

|  | **Cohort I** | | | **Cohort II** | | |
| --- | --- | --- | --- | --- | --- | --- |
| **Clinical Outcomes** | | | | | | |
|  | **CD - CL (%)** | **95% CI (%)** | **p-value** | **CD-CL (%)** | **95% CI (%)** | **p-value** |
| Blood Transfusion | -0.004 | -0.273 ~ 0.263 | 0.97 | 0.04 | -0.31 ~ 0.39 | 0.83 |
| Bleeding | 0.16 | -0.28 ~ 0.61 | 0.48 | 0.14 | -0.42 ~ 0.69 | 0.64 |
| 30-day Readmission | -0.02 | -0.59 ~ 0.55 | 0.94 | -0.17 | -0.79 ~ 0.45 | 0.59 |
| ICU visit | 0.39 | 0.07 ~ 0.7 | **0.015** | 0.84 | 0.35 ~ 1.32 | **0.001** |
| **Healthcare Resource Utilization** | | | | | | |
|  | **CD - CL** | **95% CI** | **p-value** | **CD - CL** | **95% CI** | **p-value** |
| Total Costs (2023 USD) | 15 | -230 ~ 260 | 0.903 | -153 | -461 ~ 156 | 0.332 |
| Operating room time (minutes) | 27 | 24 ~ 29 | **<0.001** | 27 | 24 ~ 29 | **<0.001** |
| Length of Stay (days) | 0.00 | - 0.06 ~ 0.06 | 0.989 | -0.04 | -0.11 ~ 0.03 | 0.236 |

_Abbreviations: CD: corded ultrasonic devices; CL: cordless ultrasonic devices; SE: standard error; CI: confidence interval
*The results of the sensitivity analysis in Cohort II were inconsistent with post-PSM estimation; no difference was determined._

**Table S3:**

**Sensitivity analysis: Post GLM adjusted clinical outcomes and healthcare resource utilization by corded and cordless ultrasonic devices**

|  | **Cohort I** | | | **Cohort II** | | |
| --- | --- | --- | --- | --- | --- | --- |
| **Clinical Outcomes** | | | | | | |
|  | **CD,  %** | **CL, %** | **p-value** | **CD,  %** | **CL, %** | **p-value** |
| Blood Transfusion | 0.4 | 0.5 | 0.91 | 0.5 | 0.5 | 0.86 |
| Bleeding | 0.9 | 0.9 | 0.85 | 1.0 | 1.1 | 0.56 |
| 30-day Readmission | 1.8 | 1.7 | 0.89 | 1.7 | 1.7 | 0.97 |
| ICU visit | 0.6 | 0.3 | **0.005** | 0.7 | 0.4 | **0.038** |
| **Healthcare Resource Utilization** | | | | | | |
|  | **CD Mean (SE)** | **CL Mean (SE)** | **p-value** | **CD Mean (SE)** | **CL Mean (SE)** | **p-value** |
| Total Costs (2023 USD) | $13,127 ($36) | $13,280 ($105) | 0.16 | $13,072 ($40) | $13,471 ($123) | 0.001* |
| Operating room time (minutes) | 123 (0.32) | 108 (0.66) | **<0.001** | 120 (0.36) | 111 (0.84) | **<0.001** |
| Length of Stay (days) | 1 (0.01) | 1 (0.02) | 0.46 | 1 (0.01) | 1 (0.03) | 0.47 |

_Abbreviations: GLM: generalized linear model; CD: corded ultrasonic devices; CL: cordless ultrasonic devices; SE: standard error;
*The results of the sensitivity analysis in Cohort II were inconsistent with post-PSM estimation; no difference was determined._

**Table S4:**

**Sensitivity analysis: Post GLM adjusted differences of outcomes between CD and CL**

|  | **Cohort I** | | | **Cohort II** | | |
| --- | --- | --- | --- | --- | --- | --- |
| **Clinical Outcomes** | | | | | | |
|  | **CD - CL (%)** | **95% CI (%)** | **p-value** | **CD-CL (%)** | **95% CI (%)** | **p-value** |
| Blood Transfusion | 0.00 | -0.3 ~ 0.3 | 0.91 | 0.00 | -0.3 ~ 0.3 | 0.86 |
| Bleeding | 0.00 | -0.3 ~ 0.4 | 0.85 | -0.1 | -0.5 ~ 0.3 | 0.56 |
| 30-day Readmission | 0.00 | -0.4 ~ 0.5 | 0.89 | 0.00 | -0.5 ~ 0.5 | 0.97 |
| ICU visit | 0.4 | 0.2 ~ 0.6 | **0.005** | 0.3 | 0.1 ~ 0.6 | **0.038** |
| **Healthcare Resource Utilization** | | | | | | |
|  | **CD - CL** | **95% CI** | **p-value** | **CD - CL** | **95% CI** | **p-value** |
| Total Costs (2023 USD) | $-153 | $-367 ~ $61 | 0.16 | $-399 | $-647 ~ $-152 | 0.001* |
| Operating room time (minutes) | 16 | 14 ~ 17 | **<0.001** | 9 | 7 ~ 11 | **<0.001** |
| Length of Stay (days) | 0.02 | -0.03 ~ 0.06 | 0.46 | -0.02 | -0.08 ~ 0.04 | 0.47 |

_Abbreviations: GLM: generalized linear model; CD: corded ultrasonic devices; CL: cordless ultrasonic devices; SE: standard error; CI: confidence interval
*The results of the sensitivity analysis in Cohort II were inconsistent with post-PSM estimation; no difference was determined._
